# Nursing protects honeybee larvae from secondary metabolites of pollen

**DOI:** 10.1098/rspb.2017.2849

**Published:** 2018-03-21

**Authors:** Matteo A. Lucchetti, Verena Kilchenmann, Gaetan Glauser, Christophe Praz, Christina Kast

**Affiliations:** 1 Agroscope, Swiss Bee Research Centre, Schwarzenburgstrasse 161, 3003 Bern, Switzerland; 2 Institute of Biology, University of Neuchâtel, Rue Emile-Argand 11, 2000 Neuchâtel, Switzerland; 3 Neuchâtel Platform of Analytical Chemistry, University of Neuchâtel, Rue Emile-Argand 11, 2000 Neuchâtel, Switzerland

**Keywords:** *Apis mellifera*, pollen secondary compounds, pyrrolizidine alkaloids, *Echium vulgare*, hypopharyngeal secretions, honeybee larvae

## Abstract

The pollen of many plants contains toxic secondary compounds, sometimes in concentrations higher than those found in the flowers or leaves. The ecological significance of these compounds remains unclear, and their impact on bees is largely unexplored. Here, we studied the impact of pyrrolizidine alkaloids (PAs) found in the pollen of *Echium vulgare* on honeybee adults and larvae. Echimidine, a PA present in *E. vulgare* pollen, was isolated and added to the honeybee diets in order to perform toxicity bioassays. While adult bees showed relatively high tolerance to PAs, larvae were much more sensitive. In contrast to other bees, the honeybee larval diet typically contains only traces of pollen and consists predominantly of hypopharyngeal and mandibular secretions produced by nurse bees, which feed on large quantities of pollen-containing bee bread. We quantified the transfer of PAs to nursing secretions produced by bees that had previously consumed bee bread supplemented with PAs. The PA concentration in these secretions was reduced by three orders of magnitude as compared to the PA content in the nurse diet and was well below the toxicity threshold for larvae. Our results suggest that larval nursing protects honeybee larvae from the toxic effect of secondary metabolites of pollen.

## 1. Introduction

Over the course of evolution, plants have developed a wide array of chemical defences against herbivores [1,2], including an impressive diversity of secondary metabolites. In turn, herbivores have responded with numerous adaptations, such as enzymatic metabolism and sequestration of toxins [3]*.* Bees are a special case among insect herbivores, as they do not consume foliar tissues but feed exclusively on pollen and nectar [4]. Since secondary metabolites are not only found in leaves but are also commonly present in pollen and nectar, bees are exposed to wide array of potentially toxic compounds. In particular, plant pollen can contain high concentrations of secondary compounds [5–9]. At times, the concentrations of such compounds are much higher in pollen than in nectar [10,11]. So far, most studies on this topic have explored the impact of secondary compounds in nectar on bees, and much less is known about how the consumption of secondary compounds of pollen affects their survival [12].

**Figure 1.**
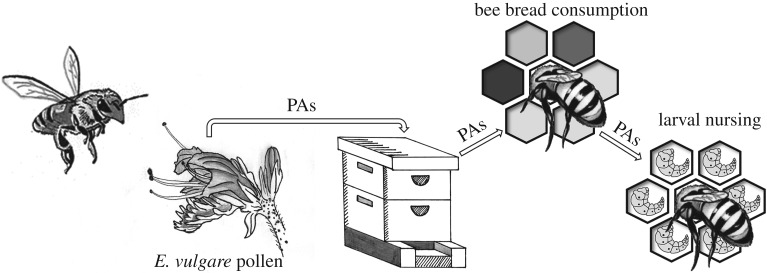
Pathway of secondary metabolites from the pollen of *E. vulgare* into bee bread and larval diets. *E. vulgare* pollen containing PAs as secondary compounds is harvested by forager bees and stored in the hive as bee bread together with other pollen types. Newly emerged honeybees consume bee bread as a protein source for the development of their hypopharyngeal glands. Mature nursing bees consume bee bread to produce hypopharyngeal and mandibular secretions to feed larvae.

The pathway of secondary compounds from pollen into the honeybee hive suggests that both adult bees and larvae are potentially exposed to these pollen compounds (figure 1). Honeybees collect pollen from a wide variety of pollen sources [13], some of which may contain toxic secondary metabolites. Worker bees combine pollen with honey, nectar and glandular secretions and store this as bee bread in the hive [14,15]. Newly emerged bees consume large quantities of this bee bread during the first few days of life, as it is central to the growth of their hypopharyngeal glands, while mature nurse bees feed on bee bread to produce hypopharyngeal and mandibular secretions [16–20], which is the main component of larval jelly [21,22]. The composition of this jelly depends on whether the larva becomes a queen (royal jelly), a worker (worker jelly) or a drone (drone jelly), but for the first 3–4 days all larvae receive a jelly that is free or almost free of pollen [23,24]. After this period, worker larvae receive a modified jelly that is less rich in protein and contains traces of pollen [23,25–27]. Hence, honeybee larvae directly consume only small amounts of pollen containing secondary metabolites, since most of the diet is composed of jelly that is secreted by nursing bees. In striking contrast, the larvae of solitary bees and bumblebees feed on a mix of pollen and nectar and, hence, they are more directly exposed to the secondary metabolites of pollen. We therefore hypothesized that the production of nursing jelly may protect honeybee larvae against exposure to plant secondary compounds.

Secondary metabolites present in pollen may affect honeybees in various ways. For one, toxic pollen metabolites may impact the survival of newly emerged worker bees and mature nurse bees, since they consume large amounts of bee bread. This would be in line with a previous study, which suggested that almond pollen could be toxic for bees if exclusively consumed by workers for more than a week [8]. Second, the secondary metabolites of pollen may indirectly impact honeybee larvae, if these compounds are transmitted from bee bread into the nursing secretions. To explore these possibilities, we used *Echium vulgare* as a plant model. This plant is widespread in Europe, produces copious amounts of floral nectar and pollen and is extensively visited by bees. *Echium vulgare* contains pyrrolizidine alkaloids (PAs) as secondary metabolites in many tissues, including the leaves and pollen [7,11,28–30]. Particularly high PA concentrations are found in pollen, while the PA concentrations in nectar are approximately 500-fold lower [11]. Thus, the high PA content of *E. vulgare* pollen may constitute a potential risk for honeybees.

In this study, we extracted PAs from *E. vulgare* and examined the effect of these secondary compounds on honeybees. First, we supplemented adult diets with PAs in concentrations that corresponded to the naturally found PA amounts in *E. vulgare* pollen. This allowed us to determine whether secondary metabolites can impact adult survival. Second, we exposed honeybee larvae to provisions containing a range of PA concentrations, which allowed us to determine the lethal PA doses for larvae. Third, we tested our hypothesis that larval nursing substantially reduces exposure of honeybee larvae to secondary metabolites. For this, we quantified the PA levels in glandular secretions produced by nurses that had previously fed on PA supplemented bee bread. If our hypothesis is valid, the PA concentrations in the secretions will be below the lethal dose for larvae. Finally, our study provides an understanding of the potential risk that secondary metabolites of pollen pose for honeybees.

## 2. Material and methods

### (a) Extraction and purification of pyrrolizidine alkaloids from *E. vulgare*

The pollen of *E. vulgare* contains particularly high amounts of secondary metabolites. Average PA concentrations ranging from 0.9 to 24.5 mg g^−1^ have been previously measured in pollen of *E. vulgare* [7,11,29,30]. In our own study on *E. vulgare* pollen collected in Switzerland, the total PA concentrations were typically between 5.4 and 9.7 mg g^−1^ [11]. Typical PAs present in the pollen of *E. vulgare* are echimidine-*N*-oxide and echivulgarine-*N*-oxide. However, these PA *N*-oxides are at least partially metabolized into the more toxic tertiary PAs in the digestive tract of honeybees [31].

We extracted echimidine and echivulgarine from the leaves and inflorescences of *E. vulgare* collected at different locations in Switzerland (for details see electronic supplementary material). Briefly, plant material was lyophilized and extracted in methanol (HPLC grade; Sigma-Aldrich, Steinheim, Germany), and the *N*-oxides were reduced with zinc dust to tertiary bases. After acid–base liquid–liquid extraction, tertiary PAs were separated using a semi-preparative system, evaporated and lyophilized. We isolated 500 mg of echimidine at high purity (94%), while we obtained less echivulgarine (25 mg; purity 62%); these quantities reflect the natural concentrations of these PAs in inflorescences and leaves of *E. vulgare* [28]. The amount of echimidine was sufficient for all bioassays presented in this study (effects on adults, larvae, and transfer into royal jelly). However since the amount of echivulgarine was not sufficient to perform all three bioassays, we decided to include this PA only in the bioassays on larvae (see electronic supplementary material).

### (b) Toxicity of echimidine for adult honeybees

We fed newly emerged honeybee workers with pollen supplemented with echimidine to examine the effect of plant secondary compounds on honeybee adults.

The honeybee (*Apis mellifera*) colonies were located at the Swiss Bee Research Centre at Agroscope, Bern, Switzerland (46°55′49″ N, 7°25′9″ E). All colonies were treated for *Varroa* infestation and tested negative for European foul brood. For each test series, frames hosting emerging broods were selected from three different bee colonies and incubated at 35°C in frame cages. After 24 h, newly emerged honeybees were collected in a glass recipient, delicately mingled to obtain a homogeneous population, and distributed equally in Liebefeld hoarding cages [18] made of stainless steel (13 × 6 × 10 cm).

We collected 5 kg of PA-free pollen during April 2015 that we later used to prepare provisions supplemented with echimidine. Pollen loads were collected daily from four bee colonies and immediately stored at −25°C. The absence of PAs above the limit of detection (LOD) of 0.7 µg g^−1^ was confirmed by UHPLC-HRMS analysis (see §2e). Furthermore, melissopalinological analysis revealed that there was no pollen from *E. vulgare*. A polyfloral honey harvested at the end of May 2015 (before the flowering period of *E. vulgare*) was used for the preparation of the supplemented pollen provisions to mask the repellent effect of the PAs. In total, four artificial provisions were prepared by mixing 2.25 g of bee-collected pollen with 1.00 g of honey. To this pollen/honey mixture, 0.5 mg, 5 mg or 25 mg of echimidine in 62.5 µl of acetone was added; this produced provisions with echimidine concentrations of 0.15 mg g^−1^, 1.53 mg g^−1^ and 7.69 mg g^−1^, respectively. The control provision contained 62.5 µl of acetone. These concentrations corresponded to doses of 2, 20 and 100 µg bee^−1^, respectively, assuming that all 50 bees in a cage consumed the same amount of provision. The pollen provision with the highest echimidine concentration (7.69 mg g^−1^) reflected the natural PA content of *E. vulgare* pollen [7,11], while the 1.53 mg g^−1^ provision reflected feeding on *E. vulgare* pollen with a rather low PA content [29,30] or mixed pollen sources.

The amount of pollen consumed by 50 bees within the first 6 days after emergence was approximately 0.65 g, which amounts to 13 mg per bee. Aliquots of 0.65 g of the provisions were offered to 50 bees per cage at day 0. Sucrose solution (50:50, w/w) was provided *ad libitum* and replaced every 3 days. Cages were placed in an incubator set to a temperature of 30°C and 75% relative humidity (RH). Dead bees were removed and counted every day. The experiment was stopped after all the bees were dead. For each experiment, the test and control series were conducted in triplicate. In addition, the entire experiment was repeated three times (approximately 450 bees per data point).

### (c) Toxicity of echimidine for honeybee larvae

We examined the effect of echimidine at a range of concentrations on the development of honeybee larvae.

First instar larvae were obtained from three bee colonies in 2015 and in 2016. In each colony, a comb with empty cells was placed in a queen excluder cage. Three days later, the queen of each colony was confined in the queen excluder cage for 24 h. Oviposition was confirmed by visual inspection after the queen was released. After 3 days, first instar larvae were collected with a disinfected paint brush 3/0.

Larval diets A, B or C containing variable amounts of sugars, yeast extract and royal jelly were prepared according to the method of Aupinel *et al*. [32]. Sugars (d (+)-glucose anhydrous and d (−)-fructose; Merck, Darmstadt, Germany) and yeast extract (Becton, Dickinson and Company, Allschwil, Switzerland) were dissolved in MilliQ water. The solutions were filtered through a 0.2-µm mesh cellulose acetate filter (Hahnemuehle, Dassel, Germany) and combined with royal jelly (see details in table 1) previously produced at the Swiss Bee Research Centre. Echimidine was dissolved in acetone (SupraSolv; Merck, Darmstadt, Germany) and supplemented at equal concentrations in the diets A, B and C (table 2). Diets were measured as volumes and echimidine concentrations were adjusted to correct for the density increase in the larval diets from A to C (for details, see table 2). For negative controls, 10 µl of acetone was added to the diet. In total, six concentrations of echimidine (10 to 80 µg g^−1^) were prepared (table 2). Assuming that each larva would consume the entire diet, the cumulative echimidine dose consumed per larva ranged from 1.8 to 14.1 µg larva^−1^ (also listed in table 2).

**Table 1:** Composition of the diets used in the larval tests.

	day 1	day 3	day 4	day 5	day 6
diet	A	B	C	C	C
volume per larva (µl)	20	20	30	40	50
royal jelly (g)	47.6	47.0	46.6	46.6	46.6
yeast extract (g)	1.0	1.4	1.9	1.9	1.9
d(+)-glucose (g)	5.7	7.0	8.4	8.4	8.4
d(−)-fructose (g)	5.7	7.0	8.4	8.4	8.4
MilliQ H_2_O (g)	40.0	37.6	34.7	34.7	34.7
total (g)	100	100	100	100	100

**Table 2.** Echimidine concentrations in diets and the cumulative echimidine doses for honeybee larvae.

	PA conc. in the diets (µg g^−1^)	PA in diet A (µg larva^−1^)	PA in diet B (µg larva^−1^)	PA in diet C (µg larva^−1^)	PA in diet C (µg larva^−1^)	PA in diet C (µg larva^−1^)	cumulative PA over 7 days (µg larva^−1^)
volume per larva (µl)		20	20	30	40	50	160
echimidine	10	0.21	0.22	0.34	0.45	0.56	1.8
	15	0.31	0.33	0.50	0.67	0.84	2.6
	20	0.42	0.44	0.67	0.89	1.12	3.5
	30	0.62	0.65	1.00	1.34	1.67	5.3
	40	0.83	0.87	1.34	1.79	2.23	7.1
	80	1.66	1.74	2.68	3.57	4.46	14.1

A chronic exposure test series was performed on larvae according to Aupinel *et al*. [32], with minor modifications. Polystyrene grafting cells (code CNE/3; Nicoplast Society, Maisod, France) for hosting the larvae were disinfected with 70% ethanol and dried at 50°C. The cells were then transferred into 48-well tissue culture plates (Greiner Bio-One; Frickenhausen, Germany) previously filled with cotton dental rolls (Ø 8 mm; Hartmann, Neuhausen, Switzerland) soaked with 500 µl of 13.2% glycerol (Merck, Darmstadt, Germany) in 0.4% methylbenzethonium chloride (Sigma-Aldrich, Steinheim, Germany) solution. At day 1, larvae in the first instar stage were grafted with paint brushes (3/0) and placed in cells containing 10 µl of diet A without PAs. Subsequently, another 10 µl of diet containing PAs was added, such that the final PA concentration was that of diet A. The tissue culture plates containing the cells were placed in a hermetic humidity chamber (Nalgene 5314-0120; Thermo Scientific) containing a saturated solution of potassium sulphate (Emsure; Merck, Darmstadt, Germany) to maintain a RH of 95%. The chamber was placed in a 34.5°C incubator. At day 3, larvae were fed with 20 µl of diet B, while at day 4, day 5 and day 6, larvae were fed with 30, 40 and 50 µl of diet C, respectively. Larval mortality was monitored daily. Dead larvae were discarded and not replaced. Food that was not consumed by day 7 was removed prior to transferring the grafting cells into a new sterile culture plate. Subsequently, the plates were kept in a humidity chamber containing a saturated solution of sodium chloride (Merck, Darmstadt, Germany) at 70% RH. At day 15, culture plates were individually placed in plastic containers, together with a piece of honeycomb, until the bees emerged.

### (d) Tracing echimidine from bee bread into royal jelly

Lastly, we examined whether echimidine was transferred from bee bread into larval jelly. For this, we quantified the levels of echimidine found in the royal jelly produced by nurses that fed exclusively on echimidine-supplemented bee bread.

For the preparation of bee bread provisions, we harvested 400 g of PA-free bee bread in 2016. The bee bread pellets were carefully removed from the combs with a metal spatula, ensuring that no wax particles were collected. The pellets were frozen at −20°C and subsequently homogenized with an electric mill. The absence of PAs above the LOD of 1.4 µg g^−1^ was confirmed by UHPLC-HRMS analysis (see §2e). For PA addition, 120 mg of echimidine was dissolved in 1 ml of acetone and mixed with 60 g of bee bread, resulting in a final echimidine concentration of 2000 µg g^−1^. As a control, 60 g of bee bread was mixed with 1 ml of acetone. For the production of royal jelly, it is essential to ensure that the bees feed on large amounts of bee bread. Therefore, we used echimidine at a concentration of 2000 µg g^−1^, as this is well tolerated by adult worker honeybees. Unlike in the adult test, we could not use honey to mask the repellent effect of echimidine. Based on the known PA content in pure *E. vulgare* pollen collected in Switzerland [11], the PA content in bee bread could be two- to fivefold higher if the bee bread is exclusively derived from *E. vulgare* pollen. However, we used a PA concentration that would be similar to that found in a honeybee colony in a natural environment, since honeybees typically collect pollen from numerous plant species and *Echium* pollen gets diluted in bee bread.

We developed a modified hive system, wherein worker bees were in a closed system and could exclusively feed on echimidine-supplemented bee bread: 600 g of bees (corresponding to 5000–6000 workers; with no queen) were carefully brushed into small Miniplus^®^ hives, an experimental unit that replicates the behaviour of a full-sized colony [33]. In total, six colonies were created: three experimental colonies and three controls. The Miniplus systems were modified such that an external cage (30 × 20 × 30 cm) with wooden sides and covered with a fine metal net was screwed over the entrance hole. This external cage allowed for the cleaning activities of the bees but prevented foraging and forced the nursing bees to feed on the bee bread and honey placed inside the hive system. A preliminary trial suggested that 60 g of bee bread was sufficient for the needs of each colony.

Experimental colonies received echimidine-supplemented bee bread, while the control colonies received non-supplemented bee bread. For each colony, 60 g of bee bread was pasted into the wax cells of an empty comb. Larvae in the first instar stage were obtained from three bee colonies and grafted into plastic cells fixed to queen rearing frames. The frame containing the plastic cells with the larvae and the comb hosting the bee bread, together with a comb filled with 500 g of a polyfloral spring honey and a comb filled with water, were placed into queen-less colonies in a modified Miniplus system as described above. Colonies were kept at ambient temperature in a room with natural light. After 3 days, colonies were transferred at 13°C for 3 h prior to collection of the cells containing royal jelly. Wax caps and larvae were removed from cells containing royal jelly. Cells with royal jelly were detached from the queen rearing frame and stored at −20°C. New cells, hosting newly grafted larvae, were glued to the queen rearing frame and placed back into the colony for the production of a new batch of royal jelly. The procedure was repeated every 3 days for a total of three harvests per colony. The bee bread remaining after the three harvests was weighed in order to calculate the amount of bee bread consumed per colony. The experiment was repeated independently three times, each time with an experimental and a control colony.

### (e) Quantification of echimidine in royal jelly, bee bread and bee-collected pollen, using UHPLC-HRMS analysis

For sample preparation of the bee-collected pollen, 1 mg of pollen was mixed with 100 µl of extraction solvent A (70% methanol, 29.5% ultrapure water and 0.5% formic acid, v/v) and transferred into a 2-ml Eppendorf tube. Five glass beads (Ø 2 mm; Sigma-Aldrich, Steinheim, Germany) were added, and the tube was shaken at 30 Hz for 4 min. Following centrifugation (18 400*g*, 4 min), 5 µl of the supernatant was transferred into a glass vial and diluted 10 times with the extraction solvent.

For sample preparation of the bee bread, 10 mg of bee bread was mixed with 1000 µl of extraction solvent A and transferred into a 2-ml Eppendorf tube. Five glass beads were added, and the tube was shaken at 30 Hz for 4 min. Following centrifugation (18 400*g* for 4 min), 10 µl of the supernatant was transferred into a glass vial and diluted 20 times with the extraction solvent.

For sample preparation of royal jelly, 100 mg of royal jelly was weighed using a microbalance scale (Mettler Toledo), mixed with 1000 µl of extraction solvent B (98% ultrapure water and 2% formic acid; 98% purity; Sigma-Aldrich, Steinheim, Germany) and transferred into a 2-ml Eppendorf tube. Five glass beads, were added, and the tube was shaken at 30 Hz for 4 min. Following centrifugation (18 400*g* for 4 min), the supernatant was collected and purified on a BondElute SCX SPE cartridge (1 ml; Agilent Technologies, USA). Cartridges were washed with 1 ml of methanol and conditioned with 1 ml of extraction solvent B. Samples were loaded onto the column and washed with extraction solvent B. After drying, the samples were eluted into a glass vial using ammoniated methanol [34,35] and dried at 40°C for 2 h using a centrifugal evaporator (CentriVap, Labconco). Samples were then re-dissolved in 500 µl of a 70% methanolic solution using an ultrasonic bath.

The detection and quantification of PAs in royal jelly, bee bread and bee-collected pollen by UHPLC-HRMS analysis were performed according to Lucchetti *et al*. 2016 [11]. In brief, PA analysis was performed on an Acquity BEH C18 column (50 × 2.1 mm i.d., 1.7 µm particle size, Waters), using an Acquity UPLC™ system (Waters) coupled to a Synapt G2 QTOF mass spectrometer (Waters). The injection volume was 1 µl. The QTOF was operated in the electrospray positive mode over a mass range of 50−600 Da. A leucine-enkephalin solution at 400 ng ml^−1^ was infused throughout the analysis to ensure high mass accuracy (less than 2 ppm). PAs were identified on the basis of their retention times, exact mass fragmentation and characteristics, and comparison with existing literature [7,11] and databases (Dictionary of Natural Products, CRC Press, USA, version 6.1. on DVD) containing information on known PAs in *Echium* spp. Quantification was performed by external calibration using echimidine (purity 94%) from Phytolab (Vestenbergsgreuth, Germany) as the standard. Linear responses were obtained from 5 to 4000 ng ml^−1^. For echimidine, the limit of quantitation (LOQ) was 2 ng ml^−1^ (signal-to-noise ratio (s/n) of 10) and the LOD was 0.7 ng ml^−1^ (s/n 3). This corresponded to LODs of 0.7, 1.4 and 0.0035 µg g^−1^ in pollen, bee bread and royal jelly, respectively.

### (f) Statistical analyses

Survival analysis of adult bees was performed with R v. 3.4.1 [36] using the survival [37] and the coxme package [38]. The data were analysed using a mixed effects Cox model [39,40], with the 36 cages, each containing of about 50 bees, included as random effects. No censoring was applied, since all the bees were dead at the end of the experiment and every death was a single event. The likelihood ratio test showed that random cage effects were statistically significant (*p* < 0.001). ANOVA analysis was performed using the car package [41] to identify the significance of the experiment, and echimidine concentrations were modelled as categorical factors. Finally, pairwise comparisons of the experiments and concentration levels were performed using Tukey contrasts with single-step adjustment for multiple testing with functions of the package multcomp [42].

Larval survival functions were estimated using the Kaplan–Meier estimator [43]. Pairwise log-rank tests were performed with SPSS 11 (SPSS 2005) for Macintosh OS X. Juvenile larvae that completed their development and emerged as adults were considered as censored observations. Multiple comparisons were accounted for by the application of the Bonferroni correction at a significance level *α* = 0.05 using the analysis option ‘pairwise for each stratum’.

For estimation of the median lethal dose (LD_50_), model fitting was performed with the general model fitting function *drm* of the R (v. 3.3.2) package drc [44] for analysis of concentration/dose/time-effect/response data. LD_50_ was calculated using a three-parameter log-logistics function with the lower limit set at 0. For details, see electronic supplementary material: model fitting functions for the estimation of the median lethal echimidine dose (LD_50_).

## 3. Results

### (a) Toxicity of echimidine for adult bees

**Figure 2.**
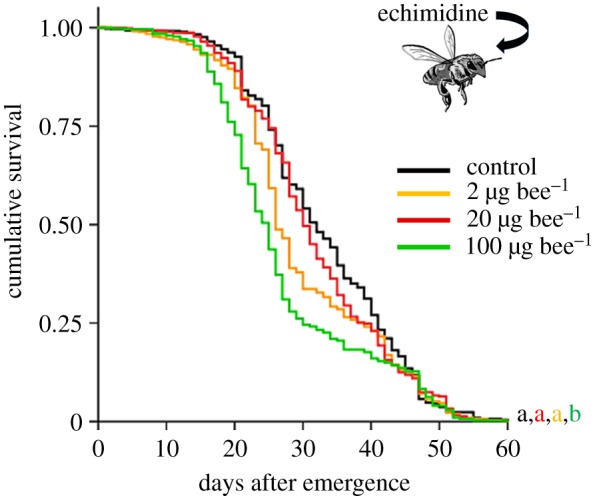
Toxicity of echimidine for adult bees. Survival curves represent the control group (*n* = 459), bees fed with echimidine at 2 µg bee^−1^ (*n* = 451), 20 µg bee^−1^ (*n* = 455) and 100 µg bee^−1^ (*n* = 448). The results for each concentration are reported as the median values of three experiments. For each experiment, test and control series were performed in triplicate. Letters at the end of the curves designate significant differences between the treatment groups (pairwise comparisons of means, *p* < 0.02).

To assess the mortality risk of adult bees after they consumed secondary metabolites from pollen, we tested the effect of echimidine supplemented in pollen provisions on newly emerged adults. Control provisions or provisions containing 2 µg, 20 µg or 100 µg echimidine per bee were consumed within 6 days. The maximal lifespan of the bees in our assays was 63 days. Little mortality was observed within the first 15 days for all the tested echimidine concentrations and controls. Thus, no acute echimidine toxicity was observed. However, the lifespan of adults fed with echimidine provisions at 100 µg bee^−1^ was shortened compared to the lifespan of bees fed with control provisions or provisions at 2 or 20 µg bee^−1^ (figure 2). The effect of the 100 µg bee^−1^ dose was significantly different from that of the control (*p* < 0.001, adjusted for multiple testing) or lower doses of 2 or 20 µg bee^−1^ (*p* < 0.02), while doses of 2 or 20 µg bee^−1^ did not show significant differences between each other (*p* > 0.8) or in comparison with the control (*p* > 0.3). Thus, echimidine had an effect on adult survival at a dose of 100 µg bee^−1^.

### (b) Toxicity of echimidine for honeybee larvae

**Figure 3.**
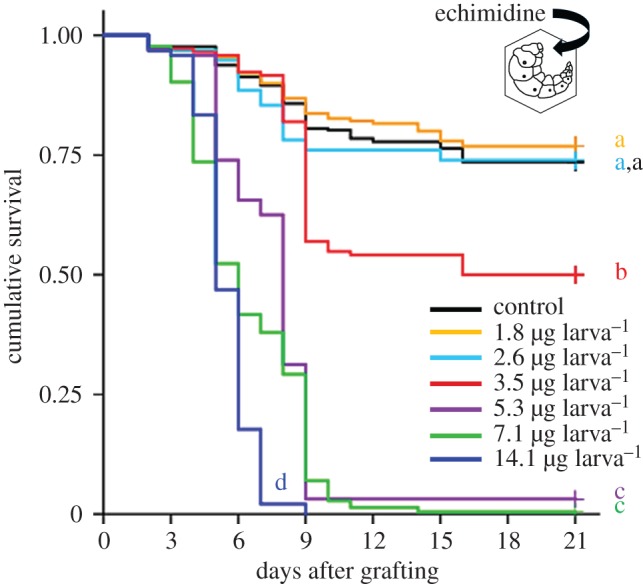
Toxicity of echimidine for larvae. Survival curves represent the control larvae (*n* = 288), larvae fed with echimidine at concentrations of 1.8 µg larva^−1^ (*n* = 190), 2.6 µg larva^−1^ (*n* = 96), 3.5 µg larva^−1^ (*n* = 144), 5.3 µg larva^−1^ (*n* = 96), 7.1 µg larva^−1^ (*n* = 216), and 14.1 µg larva^−1^ (*n* = 96). Bioassays were terminated at day 21, after the bees emerged as adults. Letters at the end of the curves designate significant differences between the treatment groups (pairwise log-rank tests, Bonferroni corrected, α* = 0.0024). At least two independent test series were performed for each concentration. Survival curves show the median values.

To assess the effects of secondary metabolites of pollen on honeybee larvae, a chronic exposure test series was performed using diets supplemented with six different concentrations of echimidine. Little mortality was observed from day 1 to day 3 for all the tested echimidine concentrations. When larvae were exposed to a cumulative dose of 14.1 µg echimidine per larva, all the larvae died within 9 days (figure 3). Echimidine at a concentration of 5.3 µg larva^−1^ induced a mortality rate of 97% up to the imago stage, while 50% of the larvae fed with a cumulative dose of 3.5 µg echimidine completed metamorphosis and subsequently emerged as adults (figure 3). No significant differences in survival up to the imago stage were observed between the control and diet conditions at a cumulative dose of 2.6 µg larva^−1^ (*α* = 0.05; pairwise log-rank test, Bonferroni corrected, α* = 0.0024) or lower doses. The emergence rates were 73% (control), 76% (1.8 µg larva^−1^) and 74% (2.6 µg larva^−1^). The median lethal dose (LD_50_) recorded on day 21 (adult emergence) [45] was 3.81 µg. In conclusion, echimidine doses from 3.5 to 14.1 µg larva^−1^ showed significant dose-related toxicity in honeybee larvae, while the dose of 2.6 µg larva^−1^, which corresponds to an echimidine concentration of 15 µg per gram of diet, was non-lethal. Chronic exposure tests were repeated with commercially available echimidine from Phytolab and provided comparable results (electronic supplementary material, figure S1).

### (c) Transfer of echimidine from bee bread into royal jelly

To determine whether secondary metabolites of pollen are transferred from bee bread into royal jelly, we measured the levels of echimidine in royal jelly produced by nursing bees that consumed bee bread supplemented with 2000 µg echimidine per gram bee bread. Echimidine concentrations in royal jelly collected from three independent harvests were on average 3.8, 2.0 and 0.6 µg g^−1^, respectively (table 3), while echimidine concentrations in the royal jelly of the control colonies were below the LOD of 0.0035 µg g^−1^. The echimidine concentrations measured in royal jelly were below 15 µg g^−1^, which is a dietary echimidine concentration that is not lethal for honeybee larvae (figure 3).

**Table 3.** Echimidine concentration in royal jelly produced by nursing bees.^a^

harvest n°	echimidine^b^ in royal jelly (µg g^−1^)	range (µg g^−1^)
1	3.8 ± 1.3	2.3–6.9
2	2.0 ± 0.2	1.9–2.3
3	0.6 ± 0.3	0.3–1.0

^a^The nursing bees consumed echimidine provided at a concentration of 2000 µg per gram of bee bread.

^b^The average echimidine concentrations for the first (*n* = 10), second (*n* = 3) and third (*n* = 9) harvest are reported as mean ± s.d.

Experimental colonies consumed on average 35.4 ± 20.9 g of bee bread and control colonies 41.8 ± 15.1 g of bee bread. Nursing bees of experimental colonies produced on average 299 ± 102 mg of royal jelly per cell and bees of control colonies produced 261 ± 77 mg of royal jelly per cell.

## 4. Discussion

Our results indicate that PAs affected the survival of both adults and larvae, but that larvae showed much higher sensitivity to PAs than adults. However, feeding on glandular secretions protected larvae from excessive exposure to such toxins, as only a very small fraction of the pollen PAs was transmitted into larval food. To our knowledge, our study is the first to show that larval nursing overcomes the toxic properties of plant pollen.

Pollen is an almost exclusive source of protein for honeybee workers [13]. Consequently, the impact of secondary compounds of pollen is potentially important for newly emerged workers, which feed on large quantities of bee bread for the development of their hypopharyngeal glands. Our study indicates that echimidine (7.7 mg g^−1^ provision; 100 µg bee^−1^), when provided at a concentration that reflects the total PA content of pure *E. vulgare* pollen in nature [11], shortened the lifespan of newly emerged honeybees, while lower concentrations (1.5 mg g^−1^ pollen; 20 µg bee^−1^, or lower) did not affect longevity. In the natural environment, however, this effect may not be relevant for honeybee colonies, since honeybees typically collect pollen from numerous plant species [13] that they mix and store as bee bread. Consequently, in bee hives, PA-containing pollen is expected to be diluted with nontoxic pollen. Additionally, high PA concentrations may have a deterrent effect on honeybees [31,46], and such a deterrent effect may further reduce the exposure of adults to pollen toxins. The relatively high tolerance of honeybee workers to PAs is in agreement with the findings of a previous study in which adult bees were fed with sucrose solutions containing monocrotaline and a mixture of PAs isolated from *Senecio vernalis* and no acute toxicity was observed within 48 h at PA levels less or equal 50 µg bee^−1^ [31].

Contrary to adult bees, larvae were extremely sensitive to PAs. Lethal effects were observed at an LD_50_ of 3.81 µg per larva for echimidine. Similar effects on larvae were also observed for echivulgarine (LD_50_ of 12.53 µg, electronic supplementary material, figure S2 and table S1), a PA especially abundant in *E. vulgare* pollen, showing that echimidine and echivulgarine are both toxic to larvae at comparable concentrations. Within the bee hive, however, honeybee larvae are barely exposed to pollen, since the main protein source for larvae is the protein-rich glandular secretions of nurse bees. Previous studies have estimated that the amount of pollen consumed by a worker larva until the imago stage corresponds only to 5% of the entire protein amount consumed by a honeybee larva [24]. Remarkably, the larval diet of honeybees contains no pollen or only trace amounts of pollen during the first 3 days of their development [23,24], when larvae are, according to our study, more sensitive to PAs than at later developmental stages (electronic supplementary material, figure S3).

Pollen consumption may still have important implications for larval development, if the PAs present in bee bread were transmitted into glandular secretions for larval feeding. Our final experiment shows that the PA concentration in royal jelly was reduced by about three orders of magnitude as compared to the PA concentration that was supplemented in bee bread. This indicates that only a very small fraction of the pollen PAs present in the consumed bee bread passes into the larval jelly. Interestingly, the mean echimidine concentration (2.1 µg g^−1^) measured in royal jelly (table 3) was markedly below the dietary concentration (15 µg g^−1^) that was found to be non-lethal to larvae (figure 3, table 2). Taken together, the findings indicate that honeybee colonies in a natural environment are affected by the secondary compounds of *E. vulgare* pollen only to a small extent, since the larval diet contains remarkably little pollen and only a very small fraction of PAs are transmitted from bee bread into nursing secretions.

The evolution of food-producing glands in eusocial bees has been previously suggested to have several benefits. First, glandular feeding allows for more rapid larval maturation than a pollen diet, and consequently, substantially more rapid colony development [47]. Second, larval jelly is at least 90% digestible, and larvae fed with glandular secretions generate less faeces than larvae fed with pollen. The reduced amount of faeces lowers the need for clean-up operations for worker bees and is probably better in terms of hygiene in the brood cells [47]. Third, glandular secretions have antimicrobial properties and hence reduce the risk of larval infections [48]. Fourth, we propose that larval nursing protects honeybees from the toxic properties of pollen and therefore allows them to make use of the broad pollen spectrum that is typical of honeybees [13].

Our results also have important implications for our understanding of pollen utilization by bees. In contrast to honeybees, the larvae of solitary bees and bumblebees directly consume pollen and nectar provisions, and thus, are directly exposed to the secondary compounds of pollen. Our toxicity tests on honeybee larvae demonstrate that secondary compounds in pollen have the potential to strongly impact larval survival. These results are in agreement with previous studies, which show that *Echium* pollen provisions can be toxic to solitary bee larvae [49,50] and a recent study on the effect of *Lupinus* pollen alkaloids on bumblebee colony development [9]. A growing number of studies on solitary bees also suggest that many pollen types exhibit properties that hamper larval development on non-host pollen [49,51–54].

Finally, while our study focused on plant secondary metabolites, it is worth mentioning that other chemicals present in pollen, in particular pesticides, may follow a similar route from flowers to bee bread and from there into glandular secretions. The current OECD guidelines for testing pesticides on honeybees *prior* to legislation focus on worker and larval toxicity, without considering whether these compounds are transmitted into larval jelly [45]. Hence, our experimental system may serve as a model for evaluating types of chemicals that pass into larval diet and hence the chemicals to which honeybee larvae are exposed.

## Supplementary Material

Toxicity of pyrrolizidine alkaloids from Echium vulgare for honeybee larvae

## Data accessibility

Electronic supplementary material, figure S1 shows the toxicity of commercial echimidine for larvae. Electronic supplementary material, figure S2 shows the toxicity of echivulgarine for larvae. Electronic supplementary material, figure S3 demonstrates that larvae are most sensitive to PAs during the first 3 days of their development (comparison of the toxicity of echimidine for larvae when provided at day 1 or at day 3). Electronic supplementary material, table S1 lists the tested echivulgarine concentrations in diets and the cumulative echivulgarine doses for honeybee larvae. In addition, the model fitting functions for the estimation of the median lethal echimidine and echivulgarine dose (LD_50_) are provided in the electronic supplementary material. Finally, the method used for extraction and purification of echimidine and echivulgarine from *E. vulgare* is also uploaded as electronic supplementary material.
