# Supplementary material for: Nursing protects honeybee larvae from secondary metabolites of pollen
Source: Proc Biol Sci. 2018 Mar 21;285(1875):20172849. doi: 10.1098/rspb.2017.2849 (PMC5897640; doi:10.1098/rspb.2017.2849)
Supplement: Toxicity of pyrrolizidine alkaloids from Echium vulgare for honeybee larvae [file rspb20172849supp1.pdf]

Supplementary Material

Proceedings of the Royal Society B

Nursing protects honeybee larvae from secondary metabolites of pollen

Matteo A. Lucchetti[1,2], Verena Kilchenmann[1], Gaetan Glauser[3], Christophe Praz[2▪], Christina Kast[1▪,*]

[1]Agroscope, Swiss Bee Research Centre, Schwarzenburgstrasse 161, 3003 Bern, Switzerland.

[2]Institute of Biology, University of Neuchâtel, Rue Emile-Argand 11, 2000 Neuchâtel, Switzerland.

[3]Neuchâtel Platform of Analytical Chemistry, University of Neuchâtel, Rue Emile-Argand 11, 2000 Neuchâtel, Switzerland.

▪Authors contributed equally to the project.

*Corresponding author: christina.kast@agrocope.admin.ch

This Supplementary Material contains 9 pages: 3 figures, 1 table, 2 model fitting functions for the calculation of the LD_{50} and the description of the method used for the extraction of the PAs.

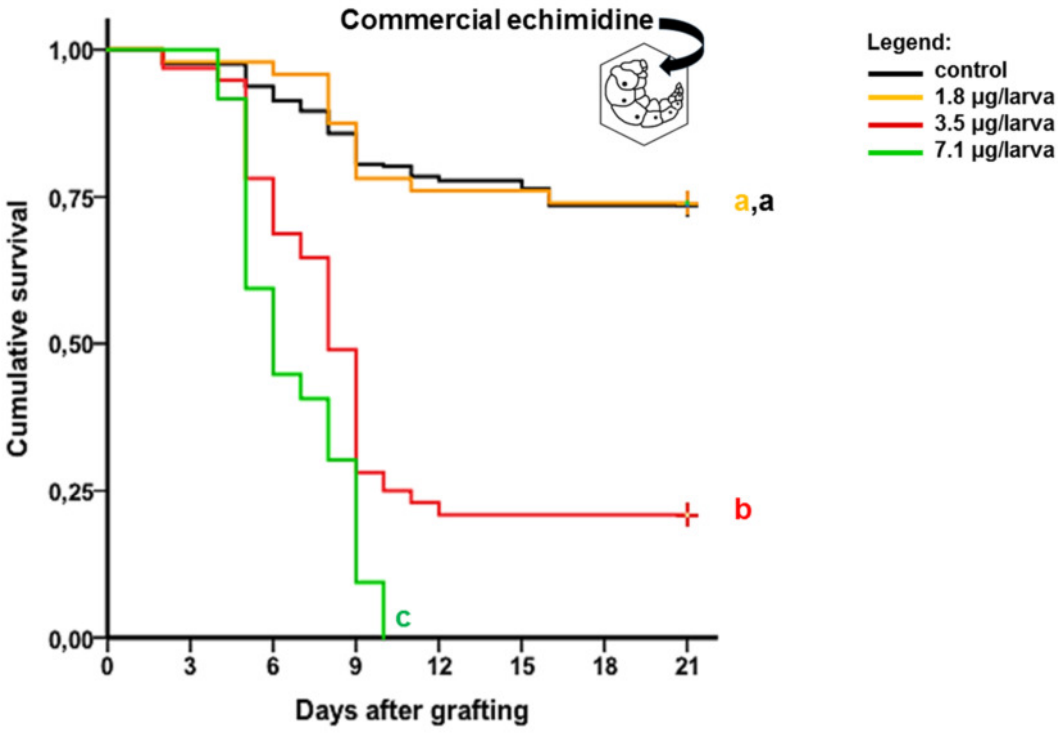

Figure S1 Toxicity of commercial echimidine (purity 94%; Phytolab, Vestenbergsgreuth, Germany) for larvae. Survival curves represent the control larvae (n=335), the larvae fed with echimidine at concentrations of 1.8 µg/larva (n=96), 3.5 µg/larva (n=96) and 7.1 µg/larva (n=96). Bioassays were terminated at day 21, after the bees emerged as adults. Letters at the end of the curves designate significant differences between the treatment groups (pairwise log-rank tests, Bonferroni corrected, $\alpha^* = 0.0083$). At least two independent test series were performed for each concentration. Survival curves show the median values.

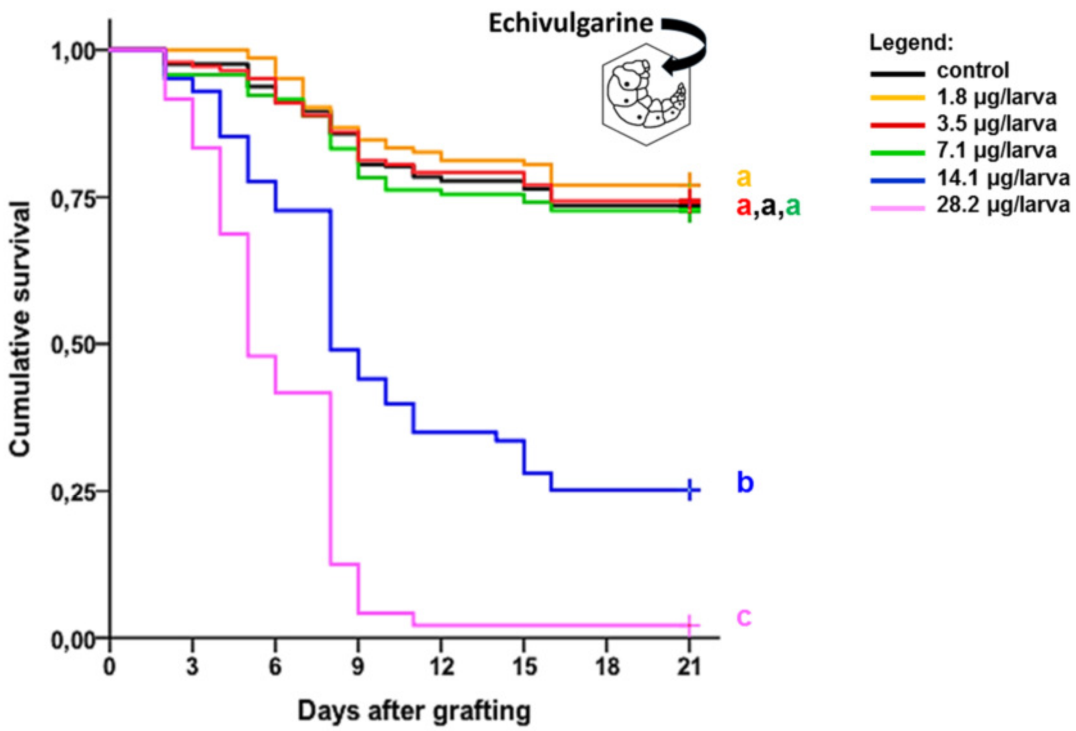

Figure S2 Toxicity of echivulgarine for larvae. Survival curves represent the control larvae (n=288), the larvae fed with echivulgarine at concentrations of 1.8 µg/larva (n=144), 3.5 µg/larva (n=143), 7.1 µg/larva (n=144), 14.1 µg/larva (n=143) and 28.2 µg/larva (n=48). Bioassays were terminated at day 21, after the bees emerged as adults. Letters at the end of the curves designate significant differences between the treatment groups (pairwise log rank test, Bonferroni corrected, α*=0.0033). At least two independent test series were performed for each concentration. Survival curves show the median values. Toxicity assays were performed as described for echimidine. The echivulgarine concentrations in the diets and the cumulative echivulgarine doses for honeybee larvae are listed in Table S1.

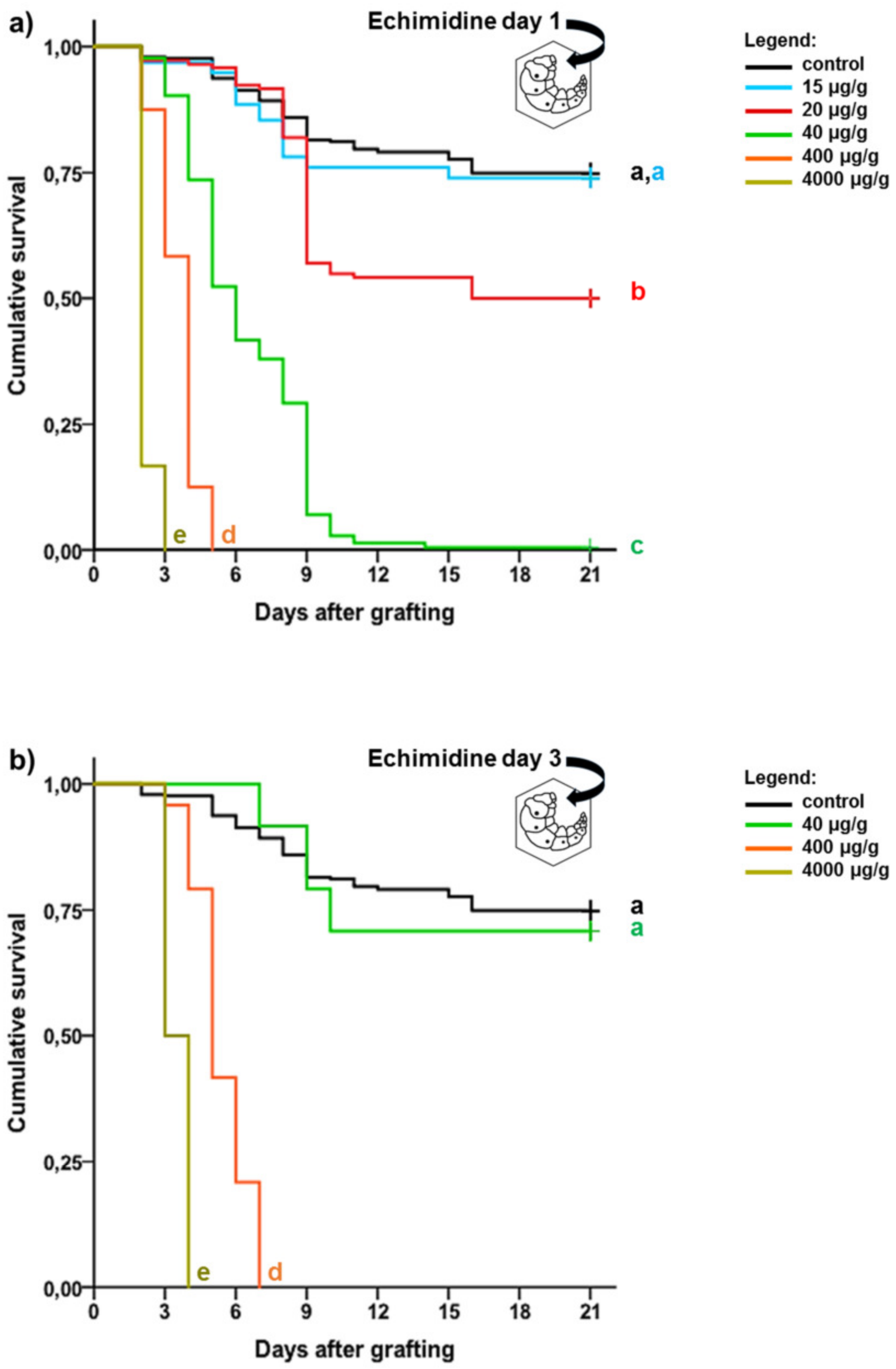

Figure S3 Toxicity of echimidine for larvae **a)** fed with a diet containing echimidine starting at day 1 or **b)** fed with a diet containing echimidine starting at day 3. Survival curves represent the control larvae (day 1: n=335; day 3: n=335), the larvae fed with a diet containing echimidine at concentrations of 15 µg/g (day 1: n=96), 20 µg/g (day 1: n=144), 40 µg/g (day 1: n=216; day

3: n=24), 400 µg/g (day 1: n=24; day 3: n=24) and 4000 µg/g (day 1: n=24; day 3: n=24). Bioassays were terminated at day 21, after the bees emerged as adults. Letters at the end of the curves designate significant differences between the treatment groups (pairwise log-rank tests, Bonferroni corrected, α^* = 0.0033 and α^* = 0.0083, respectively). At least two independent test series were performed for each concentration. Survival curves show the median values.

Table S1 Echivulgarine concentrations in the diets and the cumulative echivulgarine doses for honeybee larvae.

	**PA conc. in the diets (µg/g)**	**PA in Diet A (µg/larva)**	**PA in Diet B (µg/larva)**	**PA in Diet C (µg/larva)**	**PA in Diet C (µg/larva)**	**PA in Diet C (µg/larva)**	**Cumulative PA over 7 days (µg/larva)**
**Volume per larva (µL)**		**20**	**20**	**30**	**40**	**50**	**160**
	10	0.21	0.22	0.34	0.45	0.56	1.8
	20	0.42	0.44	0.67	0.89	1.12	3.5
**Echivulgarine**	40	0.83	0.87	1.34	1.79	2.23	7.1
	80	1.66	1.74	2.68	3.57	4.46	14.1
	160	3.33	3.49	5.36	7.14	8.93	28.2

Extraction and purification of echimidine and echivulgarine from *Echium vulgare*

Chemicals: Methanol (technical grade) was purchased from Thommen-Furler (Rüti bei Büren, Switzerland). Methanol (HPLC grade), formic acid (purity 98%), zinc dust (purity ≥ 98%), sodium chloride (purity ≥ 99%), acetonitrile (HPLC grade), ammonium hydroxide solution (28% in water, purity ≥ 99%), celite filter aid treated with sodium carbonate and flux calcined were all from Sigma-Aldrich (Steinheim, Germany). MilliQ water was obtained from a Millipore system. Sodium sulfate anhydrous was purchased from Fluka Chemie (Buchs, Switzerland), and dichloromethane from Honeywell (Seelze, Germany). 270 mm filters were obtained from J.C. Binzer (Hatzfeld, Germany).

Plant specimens of *E. vulgare* were collected at various locations in Switzerland (Basel, Bern, Thun and Verzasca valley), at various time points during the blooming season of *E. vulgare* from June until August 2013 and 2014. Specimens were collected by grasping the base of the plant with a gloved hand and pulling the closed hand up the stem to collect the majority of the inflorescences (stamens, pistils, petals and sepals) and leaves of the plant. Specimens were preserved with dry ice after collection, subsequently stored at -80°C and later lyophilized for 48 h. The dry stock (1 kg) was milled (Frittsch pulverizette), and extracted with 6 L technical methanol under continuous stirring for 24 h at 25°C. The supernatant was collected and filtered on a 270 mm paper filter, while the pellet was re-suspended in 3 L methanol and extracted one more time. Supernatants were combined and the solvents evaporated. To separate the chlorophylls, the dry extract (139 g) was reconstituted in 600 mL of a solution of 39.5% methanol, 60% water and 0.5% formic acid (v/v), stirred in an ultrasonic bath for 60 min and filtered. PA *N*-oxides were then reduced to tertiary bases with zinc dust [55]. After conversion, zinc dust was filtered out with celite powder on a membrane. An acid-base liquid-liquid extraction was performed on the PA-containing solution. For this, the pH was adjusted to 9.5 with ammonium hydroxide and dichloromethane (approximately one third of the volume of the PA-containing solution) was added together with the PA-containing solution in a separator funnel. Saturated sodium chloride was added to increase the separation efficiency. The funnel was vigorously shaken multiple times and the dichloromethane phase collected. The procedure was repeated three more times with additional dichloromethane. Sodium sulfate was added to dry the organic solution and the solvent of the combined extracts was evaporated, which yielded an oily residue. The residue was reconstituted in acetonitrile:water (70:30, v/v), divided into aliquots of 500 µL, and loaded onto a semi-preparative system composed of a 1525 EF pump (Waters, Milford, MA, USA) and a dual wavelength UV detector (2487, Waters) paired with a semi-preparative UV cell (path length 3 mm). The separation was performed on an XTerra MS C18 column (19 x 150 mm, 5 µm, Waters) using the following gradient conditions in water + formic acid 0.05% (solvent A) and acetonitrile + formic acid 0.05% (solvent B): 5% B for 3.15 min, 5-40% B from

3.15-52.27 min, 40-100% B from 52.27-59.00 min, holding at 100% B from 59.00-70.00 min, re-equilibrating at 5% B from 70.50-84.50 min. The flow rate was set as follows: 0.00-59.00 min at 8.0 mL/min, 59.50-84.00 min at 13.0 mL/min, and back to 8.0 mL/min at 84.50 min. UV detection was performed at 195 nm. The fractions were collected every minute in 13 x 100 mm glass tubes using a Gilson FC203B fraction collector. An aliquot of the fractions was re-injected in UHPLC-QTOFMS for confirmatory analysis. HPLC fractions containing exclusively either echimidine or echivulgarine were combined, evaporated and lyophilized. In total, 500 mg of echimidine and 24.6 mg of echivulgarine were isolated. The purity level of echimidine (94%) and echivulgarine (62%) was determined by UHPLC-HRMS using echimidine from Phytolab as standard. No other PA peak was detected in the purified echimidine and echivulgarine. For feeding experiments, we took into account that the purity of the extracted PAs was less than 100% (echimidine (94%) and echivulgarine (62%)). We adjusted the PA-supplementation accordingly, such that the final PA-concentrations in the diets corresponded to the concentrations listed in table 2 and table S1.

Reference

55. Crews C, Berthiller F, Krska R. 2010 Update on analytical methods for toxic pyrrolizidine alkaloids. *Anal. Bioanal. Chem*. **396**, 327–338. (doi:10.1007/s00216-009-3092-2)

Model fitting functions for the estimation of the median lethal echimidine dose (LD_{50})

```
> library(drc)

> fit <- drm( Alive/N ~ dose , na.action=na.omit,weights=N,fct = LL.3(),type="binomial")

> summary(fit)

> modelFit(fit)

> confint(fit)

> ED(fit,c(10,50),interval="delta")
```

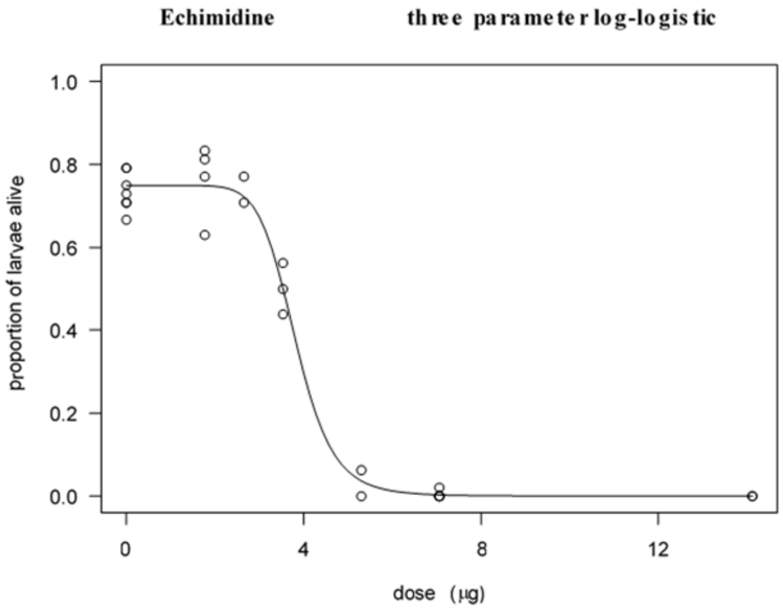

Model fitting functions for the estimation of the median lethal echivulgarine dose (LD_{50})

```
> library(drc)

> fit <- drm( Alive/N ~ dose , na.action=na.omit,weights=N,fct = LL.3(),type="binomial")

> summary(fit)

> modelFit(fit)

> neill.test(fit)

> confint(fit)

> ED(fit,c(10,50),interval="delta")
```

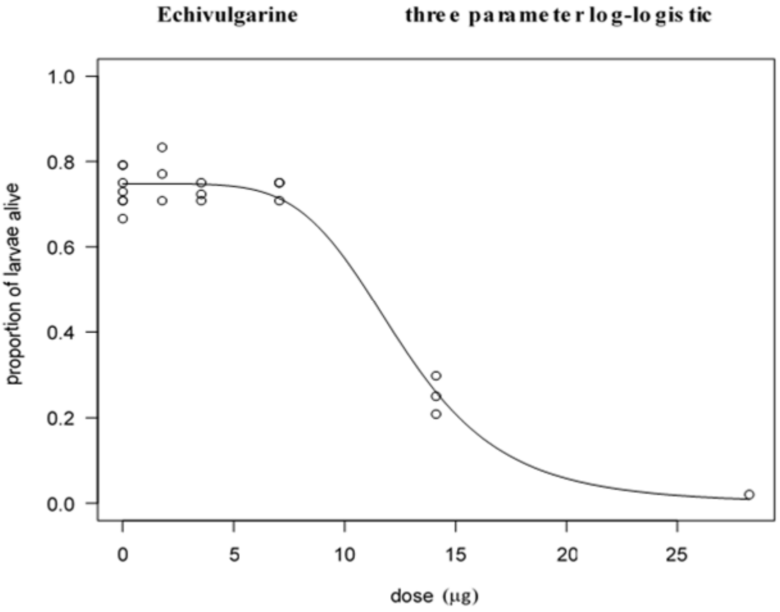
Echivulgarine
three parameter log-logistic
1.0
0.8
0.6
0.4
0.2
0.0
proportion of larvae alive
0
5
10
15
20
25
dose (μg)
